# High circulating levels of midregional proenkephalin A predict vascular dementia: a population-based prospective study

**DOI:** 10.1038/s41598-020-64998-y

**Published:** 2020-05-15

**Authors:** H. Holm, K. Nägga, E. D. Nilsson, F. Ricci, O. Melander, O. Hansson, E. Bachus, A. Fedorowski, M. Magnusson

**Affiliations:** 1 Department of Clinical Sciences, Lund University, Clinical Research Center, Malmö, Sweden; 2 Clinical Memory Research Unit, Department of Clinical Sciences Malmö, Lund University, Malmö, Sweden; 3 Institute for Advanced Biomedical Technologies, Department of Neuroscience, Imaging and Clinical Sciences, G.d’Annunzio University, Chieti, Italy; 4 Department of Internal Medicine, Skåne University Hospital, Malmö, Sweden; 5 Department of Cardiology, Skåne University Hospital, Malmö, Sweden; 6 Wallenberg Center for Molecular Medicine, Lund University, Lund, Sweden

**Keywords:** Ageing, Predictive markers, Psychiatric disorders, Risk factors

## Abstract

Midregional Pro-enkephalin A (MR-PENK A) and N-terminal Protachykinin A (NT-PTA) have been associated with vascular dementia. However, the longitudinal relationship between these biomarkers and incident dementia has not been fully investigated. In the population-based Malmö Preventive Project, circulating levels of MR-PENK A and NT-PTA were determined in a random sample of 5,323 study participants (mean age: 69 ± 6 years) who were followed-up over a period of 4.6 ± 1.6 years. The study sample included 369 patients (7%) who were diagnosed in the same period with dementia. We analyzed relationship of MR-PENK A and NT-PTA with the risk of developing dementia by using multivariable-adjusted Cox regression models adjusted for traditional risk factors. Increased plasma levels of MR-PENK A were associated with higher risk of incident vascular dementia whereas no associations were found with all-cause or Alzheimer dementia. The risk of vascular dementia was mainly conferred by the highest quartile of MR-PENK as compared with lower quartiles. Elevated levels of NT-PTA yielded significant association with all-cause dementia or dementia subtypes. Elevated plasma concentration of MR-PENK A independently predicts vascular dementia in the general population. MR-PENK A may be used as an additional tool for identifying vascular subtype in ambiguous dementia cases.

## Introduction

Dementia is a collective term for neurodegenerative diseases associated with impairment of cognitive functions including memory, speech, executive functions, and mental speed^1^. Recent epidemiological studies depict a rapid increase of individuals diagnosed with dementia from 47 million in 2015 to 131 million in 2050^2^. The most common types of dementia disorders in developed countries are Alzheimer’s and vascular dementia, accounting for more than 80% of cases^3^. Since cardiovascular comorbidities are frequently found in individuals with Alzheimer, the coexistence with vascular dementia is substantial^4^. Overlapping features of both Alzheimer and vascular dementia define mixed dementia. It is debated whether the pathophysiology of Alzheimer and vascular dementia is attributable to vascular disease, degenerative pathology, or the combination of the two. Cerebrospinal fluid biomarkers signaling neurodegenerative disease offers the opportunity to distinguish pure dementia cases from those with mixed dementia.

Since cardiovascular risk factors including hypertension, smoking, dyslipidemia, and prevalent cardiovascular disease (CVD) have been linked with dementia development^5^, biomarkers signaling cardiovascular risk have been suggested as potential diagnostic tools for cognitive impairment^6^. The stable precursor fragments of the neuropeptides enkephalin (proenkephalin A [MR-PENK A]) and substance P (protachykinin A [NT-PTA]) have been found in blood and cerebrospinal fluid (CSF)^7,8^ and have recently been used for the assessment of vascular dementia, Alzheimer’s disease and neuroinflammatory disorders^9^. In addition, high plasma levels of MR-PENK A, but not NT-PTA, have been associated with ischemic stroke and severity of cerebral injury^10^. We aimed to investigate whether elevated plasma levels of MR-PENK A and NT-PTA could be used for prediction of dementia subtypes in a community-dwelling older population.

## Methods

### Study design and population

**Figure 1:**
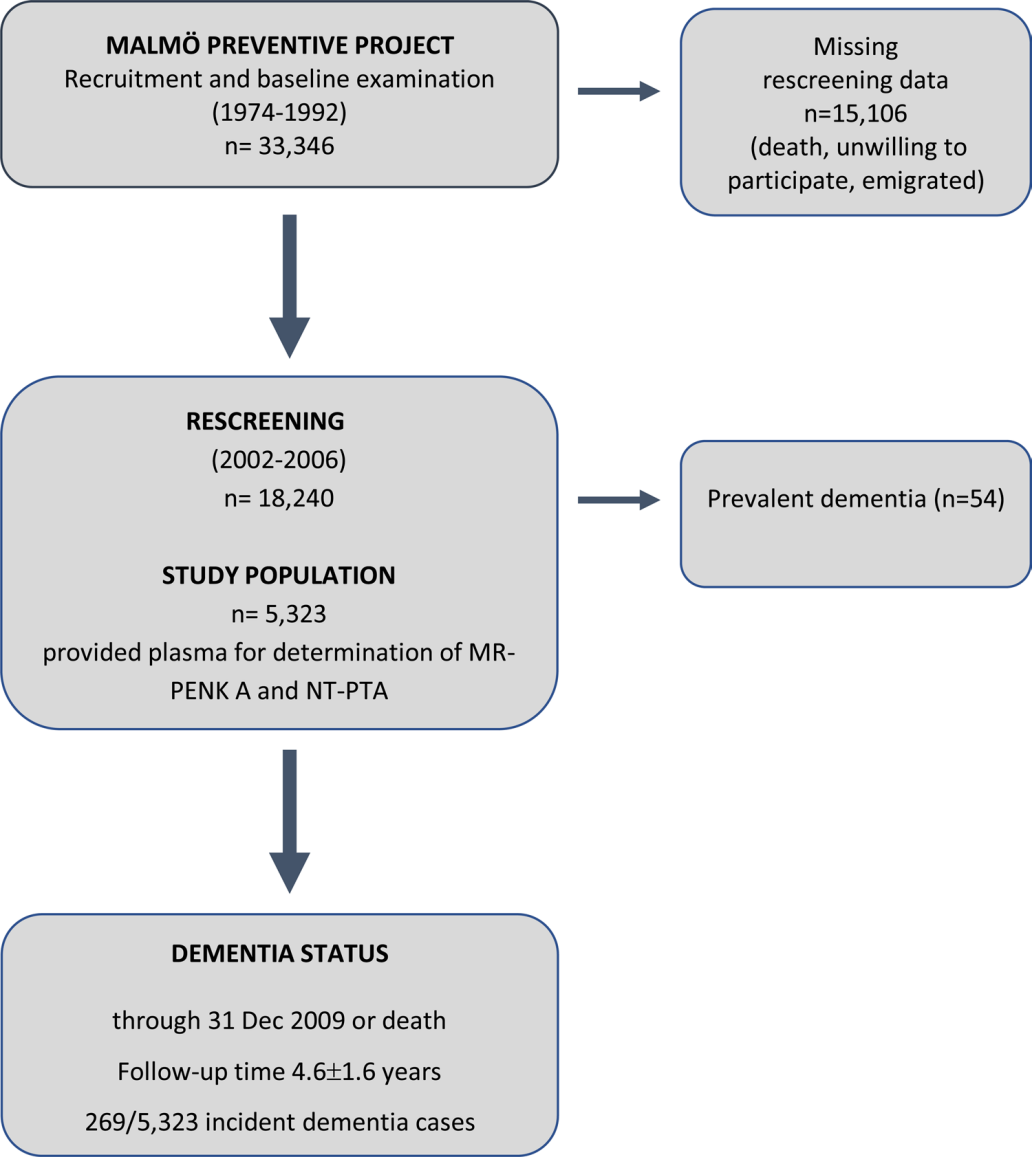
Malmö Preventive Project and re-screening program.

The Malmö Preventive Project (MPP) started in the 1970s in aim to investigate cardiovascular risk factors in 33 346 individuals living in the city of Malmo, Sweden^11^. Between 2002 and 2006, a total of 18 240 original participants responded to the invitation (participation rate, 70.5%) and were screened including a comprehensive physical examination and collection of blood samples. A through description of the project protocol has been published earlier^12,13^. In the current study, the re-examination in MPP is considered as the baseline (Fig. 1). All participants have been given an informed consent to participate and the study protocol has been approved by the Ethical Committee of Lund University, Lund, Sweden. All methods were performed in accordance with the relevant guidelines and regulations.

### Dementia diagnosis

Dementia diagnoses was retrieved from the Swedish National Patient Register (SNPR) which cover a time period between MPP baseline examination through 31 December 2009. In order to find the dementia diagnoses in the register, the International Classification of Diseases (ICD) codes 290, 293 (ICD-8), 290, 331 (ICD-9) or F00, F01, F03, G30 (ICD-10) was used. In SNPR, all in-patient care diagnoses are registered from 1987 and diagnoses from outpatient visits are covered since year 2000. The Diagnostic and Statistical Manual of Mental Disorders (DSM)-III revised edition was applied to diagnose all-cause dementia cases while the DSM-IV criteria was used to diagnose Vascular and Alzheimer’s dementia. The final dementia diagnosis was assigned by a research physician. In unresolved cases, a specialized geriatrician in cognitive disorders was consulted. Validation of dementia diagnoses was done by systematic review of medical records as well as neuroimaging. Information about dementia diagnoses retrieved from the Swedish National Patient Register (SNPR) and used in MPP has been described previously^13^. In SNPR, 471 individuals had a registered dementia diagnose and of these, 54 individuals were diagnosed before the baseline examination and was thereby excluded. Three- hundred and sixty-nine individuals were validated as incident dementia cases. Of these cases, 120 were diagnosed with Alzheimer dementia, 80 with vascular dementia, 101 with mixed type of dementia, 35 with Lewy- body dementia/Parkinson’s dementia, 4 with fronto-temporal dementia, and 29 with unspecified type.

### Biomarker assessment

Levels of MR-PENK A and NT-PTA in plasma were measured in a representative subset of 5, 309 and 5 245 participants, respectively, including all incident dementia cases. A complete dataset with both outcome information and plasma levels of MR-PENK A and NT-PTA or follow-up was available in 5 292 and 5 228 individuals, respectively. MR-PENK A is a precursor portion of the endogenous opioids methionin-enkephalin (Met-Enk) and leucine-enkephalin (Leu-Enk), while NT-PTA is the portion of the peptide precursor of substance P. MR-PENK A was assessed in fasting plasma samples that were directly frozen to −80 °C after collection. In order to estimate the level of MR-PENK A in plasma, a sensitive chemiluminometric sandwich immunoassay was used with a lower detection limit of 5.5 pmol/l. This assay acts against 119–159 amino acids of the MR-PENK A precursor fragment (BRAHMS GmbH, part of ThermoFisher Scientific, 16761 Hennigsdorf, Germany)^7,14^. A comparable chemiluminescence immunoassay was used to detect amino acids 1–37 of NT-PTA (BRAHMS GmbH, as above)^8^.

### Statistical methods

The one-way ANOVA test was used for group differences in continuous variables while Pearson’s Chi-square test was used for group differences categorical variables. Quartiles of MR-PENK A and NT-PTA plasma levels have been used for Kaplan-Meier survival analysis. Log-transformation was used as the distribution of MR-PENK A and NT-PTA was right-skewed. Cox regression model was applied to estimate hazards ratios with 95% confidence interval (CI). Log-transformed and standardized values of MR-PENK A and NT-PTA were entered as independent variables. The Cox regression model was adjusted for age, gender, systolic blood pressure (SBP), antihypertensive treatment, heart rate, smoking, diabetes, plasma cholesterol, basic education and prevalent stroke as covariates. The follow-up time was estimated as the time between baseline examination and date of dementia diagnosis, death, or end of follow-up through 31 December 2009. IBM SPSS Statistics version 23 (SPSS Inc., Chicago, IL, USA) was used to perform all analyses where p < 0.05 was considered statistically significant.

### Ethical approval

The study was approved by the Regional Ethical Review Board in Lund (LU 244-02).

## Results

Individuals with dementia were more likely to be women, older and had lower body mass index and lower BP (Table 1). The mean follow-up period from baseline examination (2002–2006) to dementia diagnosis or end of follow-up was 4.6 ± 1.6 years. Plasma concentration of MR-PENK A and NT-PTA was increased for individuals with vascular dementia compared with other dementia subtypes (Supplementary Table 1).

**Table 1:** Baseline demographic and clinical characteristics of study population (*n* = 5, 323).

Characteristics	Dementia positive (n = 369)	Dementia negative (n = 4954)	P-value
Age (years)	73 ± 5	69 ± 6	<0.001
Gender, (% male)	58	71	<0.001
Current smoker, n (%)	48 (13)	788 (16)	0.082
Supine systolic BP (mmHg)	143 ± 21	146 ± 21	0.005
Supine diastolic BP (mmHg)	81 ± 11	84 ± 11	<0.001
Heart rate (bpm)	70 ± 12	71 ± 12	0.44
Antihypertensive treatment, n (%)	157 (43)	1940 (39)	0.11
Prevalent stroke, n (%)	1 (0.3)	4 (0.1)	0.30
Plasma cholesterol (mmol/l)	5.6 ± 1.1	5.5 ± 1.1	0.60
Diabetes, n (%)	47 (13)	618 (13)	0.52
MR-PENK A (pmol/l)	66 ± 28	64 ± 27	0.129
NT-PTA (pmol/l)	86 ± 31	83 ± 28	0.029

BP, blood pressure; MR-PENK A, Midregional Proenkephalin A; NT-PTA, N-terminal Protachykinin A.

### Risk of dementia and levels of MR-PENK A and NT-PTA

**Figure 2:**
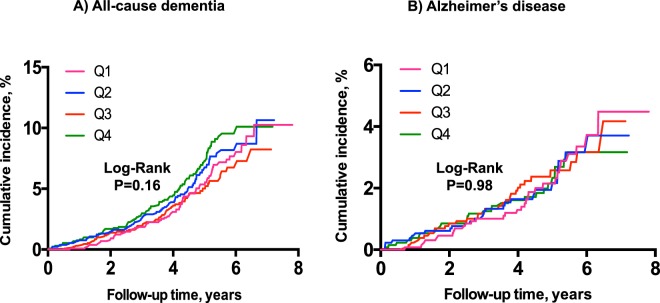
(**A,B**) Kaplan-Meier curves for cumulative incidence of (**A**) all-cause dementia and (**B**) Alzheimer disease incidence stratified according to quartiles of MR-PENK A; Q1 < 49.9 pmol/l; Q2 49.9–60.6 pmol/l; Q3 60.7–73.2 pmol/l; Q4 > 73.2 pmol/l.

**Figure 3:**
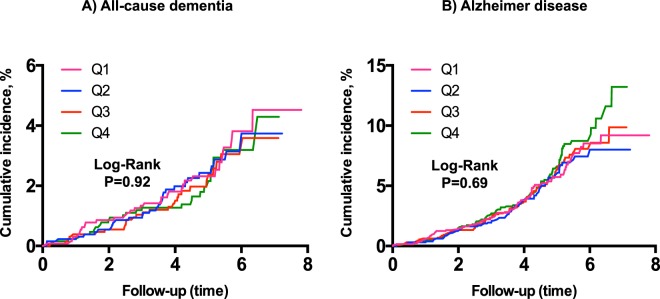
(**A,B**) Kaplan-Meier curves for cumulative incidence of (**A**) all-cause dementia and (**B**) Alzheimer disease incidence stratified according to quartiles of NT-PTA; Q1 < 66 pmol/l; Q2 66–79.2 pmol/l; Q3 79.2–94.8 pmol/l; Q4 > 94.8 pmol/l.

**Figure 4:**
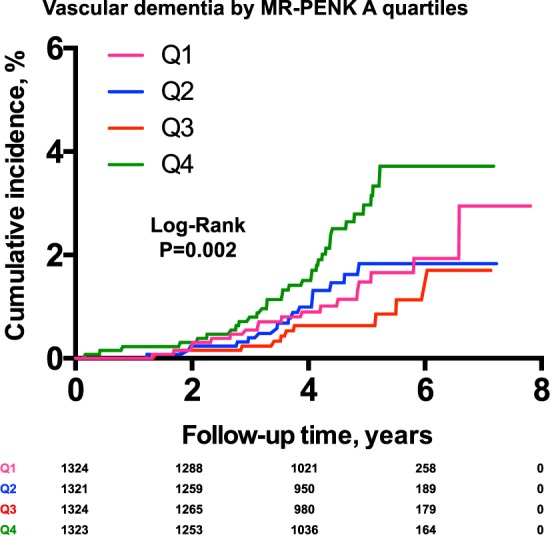
Kaplan-Meier curves for cumulative vascular dementia incidence from rescreening (2002–2006) to the end of follow-up (December 31, 2009) among 5,292 participants of Malmö Preventive Project stratified according to quartiles of MR-PENK; Q1 < 49.9 pmol/l; Q2 49.9–60.6 pmol/l; Q3 60.7–73.2 pmol/l; Q4 > 73.2 pmol/l.

As shown in Fig. 2A,B ((Kaplan-Meier curves for cumulative incidence of A) all-cause dementia and B) Alzheimer disease incidence stratified according to quartiles of MR-PENK A; Q1 < 49.9 pmol/l; Q2 49.9–60.6 pmol/l; Q3 60.7–73.2 pmol/l; Q4 > 73.2 pmol/l)) and Fig. 3A,B (Kaplan-Meier curves for cumulative incidence of A) all-cause dementia and B) Alzheimer disease incidence stratified according to quartiles of NT-PTA; Q1 < 66 pmol/l; Q2 66–79.2 pmol/l; Q3 79.2–94.8 pmol/l; Q4 > 94.8 pmol/l), quartiles of MR-PENK A and NT-PTA were not predictive of all-cause dementia or Alzheimer’s disease. However, we observed a significantly higher risk of vascular dementia in the 4^th^ quartile of MR-PENK A (values ≥ 73.2 pmol/L; log-rank test, p < 0.001) (Fig. 4; Kaplan-Meier curves for cumulative vascular dementia incidence from rescreening (2002–2006) to the end of follow-up (December 31, 2009) among 5,292 participants of Malmö Preventive Project stratified according to quartiles of MR-PENK; Q1 < 49.9 pmol/l; Q2 49.9–60.6 pmol/l; Q3 60.7–73.2 pmol/l; Q4 > 73.2 pmol/l). In the multivariable-adjusted Cox regression model, the individuals in this quartile had distinctly increased risk of vascular dementia compared with the rest of cohort (aHR 1.72, 95% CI 1.08–1.74; p = 0.02).

**Figure 5:**
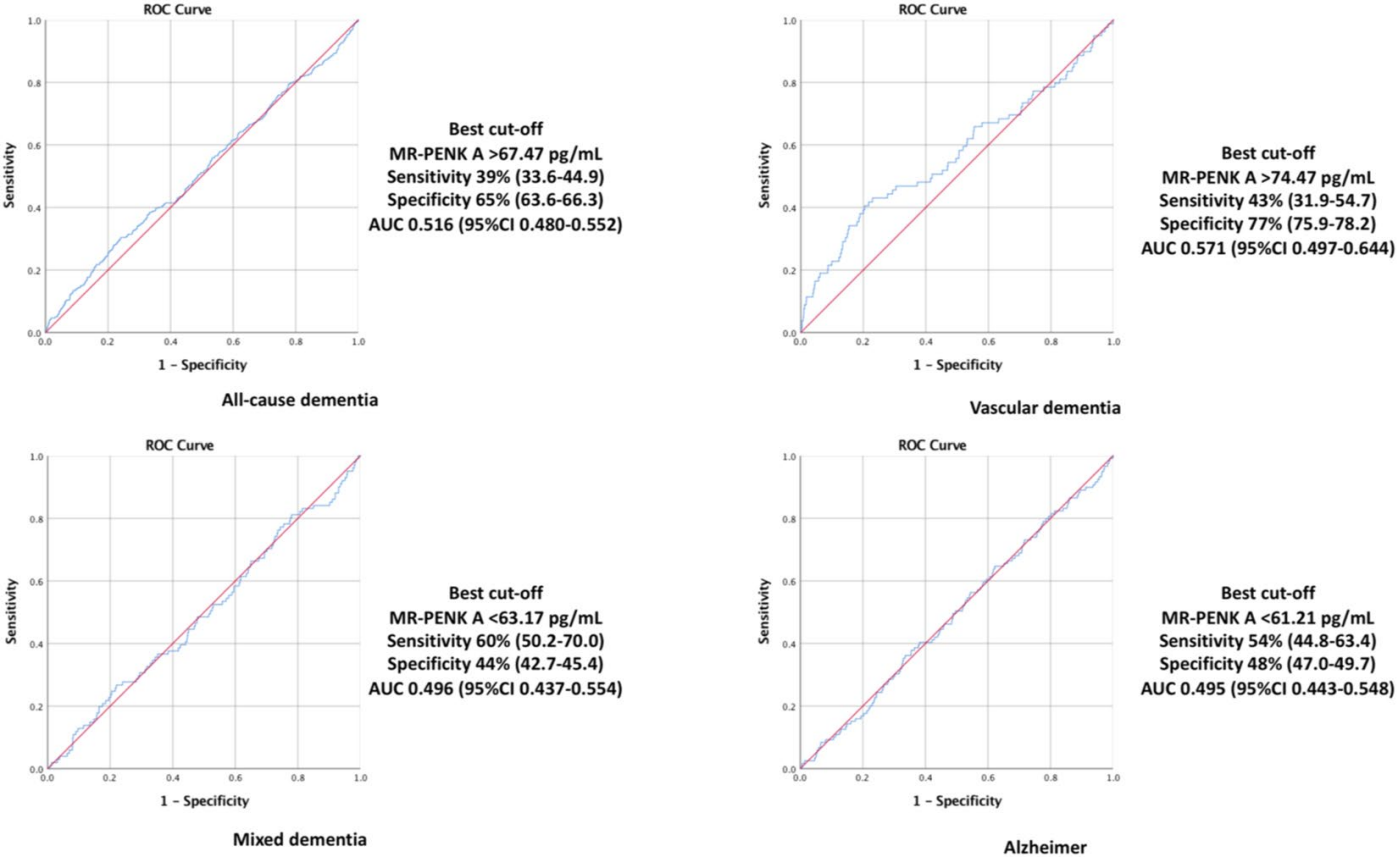
Diagnostic yield of MR-PENK A in relation to dementia subtypes by receiver operating characteristic curve analysis.

In the multivariable-adjusted model (Table 2), higher levels of MR-PENK A were significantly associated with increased risk of vascular dementia (hazard ratio (HR) per 1 SD: 1.23, 95% confidence interval (CI), 1.01–1.50; p = 0.040), whereas no significant association was observed with incident Alzheimer’s disease, all-cause dementia or mixed dementia. In ROC curve analysis MR-PENK A yielded a sensitivity of 43% (95%CI 31.9–54.7) and specificity of 77% (95%CI 75.9–78.2) for the diagnosis of vascular dementia (Fig. 5; Diagnostic yield of MR-PENK A in relation to dementia subtypes by receiver operating characteristic curve analysis).

**Table 2:** Relationship between plasma level of MR-PENK A, NT-PTA and risk of dementia in multivariable-adjusted Cox regression model.

Biomarkers	Type of dementia	
	aHR (95% CI)	P value
**All-cause dementia (n** = **369)**		
MR-PENK A	1.06(0.96–1.17)	0.246
NT-PTA	1.03(0.93–1.15)	0.553
**Alzheimer dementia (n** = **120)**		
MR-PENK A	1.06 (0.90–1.25)	0.505
NT-PTA	0.06 (0.88–1.27)	0.526
**Vascular dementia (n** = **80)**		
MR-PENK A	1.23 (1.01–1.50)	0.040
NT-PTA	1.13 (0.90–1.41)	0.290
**Mixed dementia (n** = **101)**		
MR-PENK A	0.94 (0.77–1.15)	0.544
NT-PTA	0.83 (0.68–1.03)	0.085

aHR, adjusted hazard ratio; CI, confidence interval; MR-PENK A, Midregional Proenkephalin A; NT-PTA, N-terminal Protachykinin A. Age, gender, systolic blood pressure, antihypertensive treatment, heart rate, smoking, diabetes, plasma cholesterol, basic education and prevalent stroke as covariates.

Levels of NT-PTA showed no significant association with all-cause dementia, Alzheimer disease, mixed dementia or vascular dementia (Table 2).

## Discussion

In this study, we have observed that elevated plasma concentration of mid-regional pro-enkephalin A may predict development of vascular dementia in older adults. Conversely, we have not observed any significant relationship between plasma levels of N-terminal protachykinin and incident dementia. These findings might be of importance for the understanding of the pathophysiology underlying dementia development in older adults, with particular regard to vascular subtype. Our results indicate that MR-PENK A is a promising target biomarker that may be used as an additional tool in patients with ambiguous dementia subtype diagnosis.

### A possible role of enkephalins in dementia

MR-PENK A is a stable precursor fragment and a reliable surrogate marker of the endogenous opioids methionin-enkephalin (Met-Enk) and leucine-enkephalin (Leu-Enk), which are implicated in pain sensation, cardiac function, organogenesis, immunity and ischemic tolerance^15,16^. Elevated levels of MR-PENK A have been associated with lower ejection fraction, prevalent hypertension and diabetes among heart failure patients^17^. In several studies, MR-PENK A has been identified within the CNS but also in peripheral tissues including endocrine glands, kidney, heart, lung, gastrointestinal, skeletal muscles and immune cells^15^. Enkephalins have been implicated in cognitive function, but also in the pathophysiology of dementia disorders including Alzheimer and vascular dementia^9,18^. Until recently, studies investigating the relationship between enkephalins and dementia have mainly been focused on the enkephalin concentration in the cerebrospinal fluid where reduced levels of enkephalins in individuals with Alzheimer’s disease and vascular dementia have been reported^9^. In contrast, the enkephalin concentration within the brain appears to be normal in patients with vascular dementia^18^. Since the measurement of biomarkers in plasma is less invasive than cerebrospinal fluid assessment, less costly than brain amyloid imaging and more easily measured in a primary care clinic setting, the interest in measuring plasma biomarkers for detection and prediction of dementia has increased.

There are several possible causes that might explain our findings. For example, it has been previously suggested that cognitive functions such as learning and memory are affected by enkephalin binding capacity in the CNS. A reduced binding affinity for Met- and Leu-enkephalin has been seen in AD, whereas the opioid binding in vascular dementia is still unknown^18^. In animal models, intraventricular administration of Met-enkephalin improved memory performance whereas Leu-enkephalin had no such effect^19^. Furthermore, a double-blind, placebo-controlled study showed that naloxone treatment enhanced the cognitive function in patients with AD^20^. By binding to opioid receptors, enkephalins have been proposed to be involved in ischemia pre-conditioning^21^, a mechanism recognized to protect against subsequent ischemia-reperfusion injury^22^. The presence of hypoxic ischemia is a well-known pathophysiologic factor for the development of vascular dementia^23^. Since it has previously been suggested that enkephalins are secreted from non-neuronal tissues in response to ischemia^15^, the observed increase of MR-PENK A in vascular dementia may be considered as a mechanism to restore and preserve adequate cerebral oxygenation.

MR-PENK A exerts pro-inflammatory effects, which have previously been depicted as a crucial factor for neurodegenerative changes associated with Alzheimer and vascular dementia^23^. In combination with hypoxia and oxidative stress, inflammation contributes in damaging vascular cells and oligodendrocytes^23^. By the activation of opioid receptors, enkephalins generate a depressor effect resulting in hypotension via central and peripheral receptors, causing reduced organ perfusion^24^. In this study we found that individuals with all-cause dementia had lower systolic and diastolic BP at baseline examination. This result is similar to previous reports, where BP declines within the years before clinical manifestation of dementia^25^. On the other hand, increased BP during midlife has been depicted as a central risk factor for dementia development^26^. Since PENK exerts negative inotropic effects and lower the blood pressure, the increased plasma levels of MR-PENK A observed in individuals with incident vascular dementia might be viewed as a compensatory mechanism to stimulate salt and water elimination in order to decrease the BP in the manifestation of hypertension. Nevertheless, while BP decline has CV-protective effects in healthy older individuals, it may also result in cerebral hypoperfusion, a critical factor implicated in the progression of both vascular dementia and Alzheimer’s disease^27,28^. In the current study, no significant correlation was observed between MR-PENK A and baseline systolic or diastolic blood pressure, though individuals with top quartile level of MR-PENK A were more frequently hypertensive (Q4, 42%) than subjects with lower quartiles (Q1-Q3, 38,6%) (Q4 vs Q1-Q3, Z-score = 2,235; P = 0.025). Furthermore, higher levels of MR-PENK A, but not NT-PTA have been associated with stroke severity, a well-known condition associated with vascular dementia^10^.

Dysfunction of the blood brain barrier (BBB) function may play an important role in the pathogenesis of vascular and Alzheimer dementia^29^. The BBB is a semipermeable membrane built by endothelial cells, surrounded by pericytes and astrocytes which regulates the entry of molecules from peripheral blood to the brain. During ageing, the pericytes degenerates resulting in loss of BBB integrity^30^. This leads to higher inflow of undesirable molecules entering the brain which might cause dementia. Elevated CSF concentration of soluble platelet-derived growth factor receptor-β (sPDGFRβ), a marker of vascular mural cells injury and BBB breakdown is seen in patients with mild dementia which likely reflect brain microvascular damage due to pericyte-specific injury^31^. It has previously been shown that the transport of enkephalins across the BBB is regulated by receptor-mediated transport mechanisms from blood-to-brain^32^. These findings posit that transport of encephalin across the BBB is not passive and may not reflect BBB breakdown. Since both MR-PENK A and NT-PTA are decreased in the CSF in patients with different dementia disorders^9^, dysfunction of the BBB is not likely to be the origin for the elevated levels of plasma MR-PENK A seen in the present study.

Finally, in animal models enkephalin administration induced antioxidative effects by regulation of oxidant processes and antioxidant enzyme activities^33^. Increased levels of MR-PENK A seen in individuals with incident dementia may therefore also counteract the oxidative stress, which has previously been indicated as a crucial contributor for development of vascular dementia^34^.

### Strengths and limitations

An underestimation of incident dementia is possible since primary care is not covered in the SNPR. This underestimation has been described in previous reports where the diagnoses were based on hospital discharge, not on hospital-based outpatient care^35,36^. Participants in this study were primarily of European ancestry hence the results may not be generalizable to other racial/ethnic groups. The dementia diagnoses in the present study are registry based. In the validation procedure we also looked at the CT/MRIs of the patients when available, as a part of the diagnostic validation. However, in this study we did not aim to use those data further and did not include neuroradiology or CSF as variables.

We used the clinical diagnostic criteria for dementia, but only those diagnoses of dementia that were registered in the SNPR underwent specific validation process through full review of medical records.

The association between MR-PENK A and vascular dementia was mainly driven by an increased incidence in the upper quartile, with the third quartile being associated with the lowest cumulative incidence. Therefore, only the highest plasma concentrations of MR-PENK A seem to mediate the higher risk of vascular dementia.

## Conclusion

Higher plasma concentration of MR-PENK A independently predicts vascular dementia in the general population. MR-PENK A may be used as an additional tool for identifying vascular subtype in ambiguous dementia cases. External validation is eagerly awaited before implementing such prediction models in clinical practice.

## Supplementary information

Supplementary table 1.
