# Supplementary material for: High circulating levels of midregional proenkephalin A predict vascular dementia: a population-based prospective study
Source: Sci Rep. 2020 May 15;10:8027. doi: 10.1038/s41598-020-64998-y (PMC7229155; doi:10.1038/s41598-020-64998-y)
Supplement: Supplementary file 1 — Supplementary table 1. [file 41598_2020_64998_MOESM1_ESM.pdf]

High circulating levels of midregional proenkephalin A predict vascular dementia: a population-based prospective study

H. Holm [1,5], K. Nägga [2], ED. Nilsson [2], F. Ricci [1,3], O. Melander [1,5], O. Hansson [2], E. Bachus [1,4], A. Fedorowski [1,5]*, and M. Magnusson [1,5,6]*

* Shared senior authorship.

Affiliations:

[1] Department of Clinical Sciences, Lund University, Clinical Research Center, Malmö, Sweden

[2] Clinical Memory Research Unit, Department of Clinical Sciences Malmö, Lund University, Malmö, Sweden

[3] Institute for Advanced Biomedical Technologies, Department of Neuroscience, Imaging and Clinical Sciences, G.d'Annunzio University, Chieti, Italy.

[4] Department of Internal Medicine, Skåne University Hospital, Malmö, Sweden

[5] Department of Cardiology, Skåne University Hospital, Malmö, Sweden

[6] Wallenberg Center for Molecular Medicine, Lund University, Sweden

Supplementary tables

Table S1. Baseline demographic and clinical characteristics by dementia subgroups.

Characteristics	Dementia negative (n= 4954)	Vascular dementia (n=80)	Alzheimer dementia (n=120)	Mixed Dementia (n=101)	ANOVA, P-value
**Age (years)**	69±6	74±4	73±4	74±5	<0.001
**Gender, (% male)**	71	68	42	60	<0.001
**Current smoker, n (%)**	788 (16)	16 (20)	11 (9)	15 (15)	0.161
**Supine systolic BP (mmHg)**	146 ±21	145±22	143±19	143±19	0.223
**Supine diastolic BP (mmHg)**	84±11	81±12	81±9	81±10	<0.001
**Heart rate (bpm)**	71±12	70±12	71±10	71±13	0.756
**Antihypertensive treatment, n (%)**	1940 (39)	48 (61)	39 (33)	44 (43)	<0.001
**Prevalent stroke, n (%)**	4 (0.1)	0 (0)	0 (0)	1 (1)	0.033
**Plasma cholesterol (mmol/l)**	5.6±1.1	5.2±1.2	5.8±1.1	5.7±1.2	0.001
**Diabetes, n (%)**	618 (13)	17 (20)	16 (13)	6 (6)	0.058
**MR-PENK A (pmol/l)**	64±27	74±39	63± 23	63±22	0.006
**NT-PTA (pmol/l)**	83±28	93±38	82±24	82±27	0.015

BP, blood pressure; MR-PENK A, Midregional Proenkephalin A; NT-PTA, N-terminal Protachykinin A.
